# Relational–temporal dignity at the end of life: Ethical foundations for the DiRePal model in the algorithmic age

**DOI:** 10.1017/S1478951526102648

**Published:** 2026-05-04

**Authors:** Abel García Abejas

**Affiliations:** 1 Núcleo de Estudos em Bioética (NEBUBI), Faculdade de Ciências da Saúde, Universidade da Beira Interior, Covilhã, Portugal; 2 Unidade de Medicina Geral e Familiar, Hospital Lusíadas Lisboa, Lisboa, Portugal

**Keywords:** Palliative care, dignity, ethics, artificial intelligence, patient-reported outcomes measures, narrative medicine, time perception, professional-family relations, memory, digital technology

## Abstract

**Objectives:**

The increasing presence of artificial intelligence (AI), electronic patient-reported outcomes (ePROMs), and digital infrastructures in palliative care is transforming how clinical encounters are organized and how suffering is interpreted. These technological shifts heighten the risk of relational compression and a reduction of dignity to measurable outputs. This paper proposes the DiRePal model (Relational–Temporal Dignity in Palliative Care) as a philosophical framework to re-examine dignity beyond coherent narrative identity or autonomy-centered ethics, emphasizing relational presence, temporal sensitivity, and structural conditions of care.

**Methods:**

A philosophical–ethical analysis informed by narrative identity (P. Ricoeur), ethics of alterity (E. Levinas), capabilities theory (M. Nussbaum), and care ethics (J. Tronto). Critical readings of dignity frameworks, AI ethics, and digital health literature were synthesized to develop a relational–temporal account of dignity and 2 operational concepts: the temporal dignity indicator and the architecture of prudence.

**Results:**

While digital tools can enhance communication and support anticipatory care, they also risk reducing patients to data profiles, narrowing listening practices, and eroding opportunities for narrative, silence, and relational presence. The DiRePal model reframes dignity as a fluctuating, co-constructed achievement that depends on temporal attentiveness, ethical listening, institutional conditions, and prudent integration of AI and ePROMs. It further expands dignity to include post-biographical dimensions such as memory, grievability, and digital legacy.

**Significance of results:**

End-of-life care in the algorithmic age requires an ethics that recognizes dignity as relational, temporal, and structurally mediated. The DiRePal model offers clinicians and institutions a conceptual grammar to resist technological reductionism, protect time for presence, and safeguard the narrative and post-biographical continuity of persons whose voices may be fragmented, vulnerable, or digitally extended.

## Introduction

Dignity has long been a cornerstone of medical ethics and palliative care, yet it remains conceptually contested and normatively unstable. Across centuries, it has shifted between metaphysical, moral, political, and clinical meanings. Classical traditions linked dignity to the Hippocratic injunction *primum non nocere* and to Aristotle’s *phronesis*, the prudential wisdom required to deliberate under conditions of uncertainty (Aristotle 2009). Kant (2011) later grounded dignity in intrinsic human worth, arising from autonomy and rational self-legislation, insisting that persons must always be treated as ends in themselves. Modern critiques, including those of Foucault (1980), highlighted how medical biopower and processes of normalization risk obscuring the singularity of suffering, while Canguilhem (2022) demonstrated the normative fragility inherent in distinctions between the normal and the pathological.

In palliative care, dignity is both foundational and contested. Chochinov’s dignity therapy (DT) sought to operationalize dignity through structured narrative review, demonstrating psychological benefits and enhanced legacy-building (Chochinov et al. 2005, 2011). Julião’s work, by contrast, framed dignity as an existential and symbolic reality expressed through presence, attentive listening, and the patient’s “last word” (Juliao et al. 2017, 2022). Yet both models reveal limitations: DT may reduce dignity to a coherent autobiographical product requiring cognitive capacity, while the ethics of presence, though profound, remains insufficiently embedded in systemic and technological structures (Johnston et al. 2023).

These limitations become more evident in the algorithmic age. Palliative care encounters are increasingly mediated by artificial intelligence (AI), predictive analytics, and electronic patient-reported outcomes (ePROMs). AI tools already influence referral patterns (Heinzen et al. 2023; Wilson et al. 2023), often substituting hermeneutic temporality with predictive logic (Floridi 2013; Topol 2019). While ePROMs can strengthen patient voice (Greenhalgh et al. 2018), when reduced to checklists or clinician-entered data they risk flattening existential nuance and silencing the most vulnerable (Snyder et al. 2009). The result is a form of relational compression, where encounters become shaped by screens and metrics more than by listening, presence, and time.

Philosophical resources help resist these reductions. Ricoeur’s account of narrative identity highlights the dynamic interplay between *idem* (sameness) and *ipse* (selfhood), emphasizing the need for reinterpretation and re-narration rather than static continuity (Ricoeur 1991, 1992). Levinas reframes dignity as an asymmetrical ethical responsibility toward the vulnerable Other, locating value not in internal attributes but in the ethical demand issued by the face of the patient (Lévinas et al. 1985). Nussbaum’s capabilities approach situates dignity in opportunities enabled by relational and institutional scaffolding, especially under conditions of severe dependency (Nussbaum 2011). Tronto’s ethics of care expands this further, insisting that dignity is always shaped by political and structural arrangements – attentiveness, responsibility, competence, and responsiveness (Tronto 1993).

Contemporary scholarship reinforces these insights. Brown-Johnson et al. (2019) underscore the centrality of clinician presence even in technologically saturated contexts, while Guite-Verret et al. (2023) highlight intentional presence as foundational for accompaniment in dying. Walter (1996) and Butler (2004) extend dignity into the realms of memory, grievability, and symbolic continuity. The digital era complicates this horizon further, raising concerns about the stewardship of digital remains, afterlife data, and the ethical governance of posthumous identity (Buitelaar 2017; Öhman et al. 2017, 2018).

Building on these philosophical, ethical, and technological challenges, this essay introduces the DiRePal model (Relational–Temporal Dignity in Palliative Care) as a new conceptual grammar for dignity. DiRePal reconceptualizes dignity as relationally co-constructed, temporally dynamic, and structurally mediated. It brings classical philosophical insights into dialog with 2 operational notions: the temporal dignity indicator, which considers how clinical time is allocated between presence and technological mediation, and the architecture of prudence, which embeds contestability, reversibility, and proportionality into AI-mediated systems. It further extends dignity to include a post-biographical horizon encompassing memory, grievability, and digital legacy (Walter 1996; Butler 2004; Buitelaar 2017; Öhman et al. 2018).

In this framework, dignity is understood not as an intrinsic or static attribute but as a relational, temporal, and structurally mediated phenomenon that emerges through processes of recognition, co-narration, and ethical responsiveness within clinical and institutional contexts. Accordingly, the subject of dignity is not confined to the individual patient alone but extends relationally to include families and clinicians within a shared ethical ecology of care.

The purpose of this essay is not to propose a new clinical intervention but to articulate a philosophical foundation capable of addressing the complexities of end-of-life care in technologically mediated environments. By binding presence, temporality, and structural design into a coherent ethical grammar, DiRePal offers clinicians, educators, and institutions a framework for sustaining dignity in life and in memory, even amidst the intensifying pressures of digital medicine.

This essay expands and systematizes previous work on post-biographical dignity (García Abejas et al. 2025), by developing a comprehensive relational–temporal model (DiRePal) with distinct philosophical foundations and operational tools.

## Context and critique of prevailing models

Dignity in end-of-life care has long been approached through frameworks that, while clinically valuable, often fail to capture its ethical, relational, and temporal complexity. Classical accounts – from the Hippocratic emphasis on *primum non nocere* to Kant’s understanding of intrinsic human worth – help explain why dignity remains central in medicine, yet they offer limited guidance for contemporary palliative contexts, where vulnerability, relationality, and narrative fluctuation are salient (Aristotle 2009; Kant 2011). In modern practice, dignity is challenged not only by illness but also by structural, communicational, and technological pressures that shape how individuals are recognized and cared for.

Two paradigms have been particularly influential: Harvey Chochinov’s DT and Miguel Julião’s ethics of presence. Each has expanded the discourse on dignity yet both reveal conceptual and structural limitations that underscore the need for a broader relational–temporal approach.

These 2 models are selected as paradigmatic because they represent 2 dominant and complementary approaches in contemporary palliative care: a structured narrative-operational model (DT) and an existential-relational model grounded in presence and symbolic recognition. Together, they illuminate central tensions in how dignity is conceptualized and enacted in clinical practice.

### The limits of DT

Chochinov’s DT remains the most widely used attempt to operationalize dignity in palliative care (Chochinov et al. 2005, 2012). It has demonstrated measurable benefits in reducing existential distress and promoting continuity of self through structured life review (Chochinov et al. 2011; Juliao et al. 2017). However, its underlying assumptions privilege coherent autobiographical narrative, cognitive capacity, and linear memory reconstruction, thereby excluding many of the most vulnerable patients – such as those with advanced cognitive decline, severe frailty, fluctuating consciousness, or fragmented self-expression (Johnston et al. 2023).

Furthermore, DT’s scripted structure may unintentionally reinforce fixed biographical identities. By relying on recollection rather than reinterpretation, DT can confine dignity to a stable continuity of self, insufficiently sensitive to the ruptures, ambiguities, and temporal fragility characteristic of terminal illness. This narrative dependency limits the model’s applicability in contexts where identity is episodic, relationally mediated, or expressed through silence, gesture, or presence rather than through coherent storytelling.

### The ethics of presence and its constraints

Julião’s contributions highlight the ethical and symbolic depth of dignity, grounded in attentive presence, listening, and the “last word” of the patient (Juliao et al. 2017, 2022). This approach illuminates essential dimensions of recognition and meaning-making near the end of life. Yet an exclusive focus on the interpersonal encounter risks idealizing the dyadic moment and underemphasizing the structural and organizational realities that profoundly affect dignity. Time constraints, digital documentation demands, institutional hierarchies, and technological mediation can erode the very conditions that make ethical presence possible (Tronto 1993; Brown-Johnson et al. 2019).

Presence, while indispensable, requires systemic support: protected time, relational continuity, team culture, and technological environments that do not dominate the clinical encounter. Without such scaffolding, presence becomes morally significant yet structurally fragile – too dependent on individual virtue and too vulnerable to the pressures of efficiency-driven, digitally mediated care.

### Toward a relational–temporal turn

Both DT and the ethics of presence reveal dignity’s indispensability but also its conceptual fragility. DT risks reducing dignity to a structured narrative product; presence risks relying on an interpersonal ideal insufficiently protected by institutional or technological arrangements. Neither framework fully addresses the relational, temporal, and structural forces shaping dignity in contemporary care.

What is needed is a relational–temporal reframing: a perspective that understands dignity as something co-created between patients, families, and clinicians; that fluctuates across illness trajectories; and that is mediated by organizational cultures, digital infrastructures, and modes of listening. This need becomes even more urgent in the algorithmic age, where AI, ePROMs, and predictive analytics increasingly shape clinical perception and time allocation (Floridi 2013; Topol 2019). These technologies risk compressing relational time, subordinating narrative depth to data-driven logic, and redefining vulnerability through predictive profiles.

The DiRePal model emerges in response to these challenges, offering a conceptual grammar for dignity that is relational, temporally sensitive, and structurally aware – capable of resisting technological reductionism while affirming the ethical essence of care in end-of-life settings. Existing models illuminate partial dimensions of dignity; DiRePal integrates them within a broader relational–temporal and institutional framework required for digitalized care environments.

Other approaches, including legacy-oriented and reminiscence-based frameworks, further contribute to the understanding of dignity by emphasizing continuity, meaning-making, and symbolic transmission across time. While not exhaustively reviewed here, these perspectives reinforce the importance of narrative, relationality, and memory and are conceptually integrated within the broader relational–temporal horizon proposed by DiRePal (Webster et al. 2002).

## Philosophical foundation of the DiRePal model

The DiRePal model is grounded in a relational–temporal understanding of dignity that challenges both individualistic and static accounts. Its conceptual scaffolding draws on 4 complementary philosophical traditions – Ricoeur’s hermeneutics of narrative identity, Levinas’s ethics of alterity, Nussbaum’s capabilities approach, and Tronto’s ethics of care. Together, these perspectives reposition dignity not as a possession of the autonomous subject but as a fragile, co-constructed phenomenon that emerges between persons, unfolds across time, and depends on the organizational and technological conditions in which care occurs.

### *Narrative identity and the dialectic of* idem*/*ipse *(Ricoeur)*

Ricoeur interprets identity as a dynamic narrative synthesis of *idem* (sameness) and *ipse* (selfhood), sustained through interpretative labor over time (Ricoeur 1992). This dialectic undermines attempts to reduce dignity to the preservation of a fixed biographical essence. In advanced illness, bodily decline, interruptions in routine, and existential rupture destabilize continuity of self. Dignified care must, therefore, support both recognition of what has been (*idem*) and opportunities for re-narration (*ipse*), allowing patients to reinterpret or “re-promise” themselves in the face of loss and finitude.

Crucially, this narrative orientation ensures that suffering is approached as lived experience rather than as an abstracted symptom or data point. Dignity, in this view, is preserved when the patient’s embodied vulnerability is interpreted within a relational and temporal horizon.

Three ethical implications follow. First, dignity cannot rest solely on recollection; it requires interpretive space for transformation. Second, narrative identity is mediated by listeners, witnesses, and institutional scripts – meaning dignity is co-constituted through relational practices of acknowledgement rather than authored alone. Third, clinical dignity depends on *kairotic* time – opportune, irreplaceable moments of recognition – rather than chronometric pressures of throughput. DiRePal, therefore, adopts Ricoeur’s notion of “narrative hospitality”: the disciplined readiness to host fragmented, nonlinear, or symbolic stories that sustain selfhood even when coherence falters (Ricoeur 1992; Guite-Verret et al. 2023).

### The asymmetrical ethics of the Other (Levinas)

Levinas situates ethics not in autonomy but in the encounter with the vulnerable Other, whose face calls the caregiver into responsibility prior to reciprocity or contract (Lévinas 1972). In end-of-life contexts, the Other’s fragility – often silent, dependent, or disoriented – does not diminish dignity; it intensifies its ethical claim. On this account, dignity is not exhausted by a patient’s ability to articulate a narrative but arises from the moral demand their vulnerability places upon those who care.

Levinas also highlights that caregivers are not neutral intermediaries. Their own dignity becomes implicated in responding to the Other, and this responsibility persists even under time scarcity, digital mediation, or systemic constraint. Where health systems risk reducing persons to data profiles or algorithmic risk categories, Levinasian ethics reasserts the primacy of singular encounter. For this reason, DiRePal regards recognition, attentiveness, and presence not as optional features of care but as its ethical ground (Lévinas 1972; Rushton 2018).

### Capabilities and supported agency under dependency (Nussbaum)

Nussbaum’s capabilities approach anchors dignity in the real opportunities individuals must be and to act under just conditions (Nussbaum 2011). Severe illness erodes many central capabilities – bodily integrity, expression, affiliation, and agency – yet capability loss does not equate to the loss of dignity. Dignity persists where relational and institutional scaffolding sustains opportunities for participation, decision-making, and symbolic presence.

For DiRePal, 2 consequences follow. First, dignity-preserving care requires not only attitudes, but material supports protected time for presence, communication aids, and ethically designed technologies. Second, the capabilities perspective widens the moral field to include families and clinicians. Without supporting the capability of professionals to be attentive, present, and morally resilient, patient dignity cannot reliably be upheld. The temporal dignity indicator emerges from this insight, repositioning time-for-presence as an ethical and organizational resource rather than a dispensable luxury (Brown-Johnson et al. 2019; Andersson et al. 2022).

### Care ethics and the structural conditions of dignity (Tronto)

Tronto’s ethics of care emphasizes that caring practices are shaped by attentiveness, responsibility, competence, and responsiveness, all embedded within political and institutional arrangements (Tronto 1993, 2013). This perspective exposes a blind spot in dignity frameworks that focus solely on the clinician–patient dyad: dignity is variably enabled or undermined by systemic factors such as staffing ratios, digital workflows, bureaucratic pressures, and leadership culture.

From a care-ethical lens, these institutional and technological structures must be treated as ethical determinants of dignity. Digital infrastructures can either compress relational time or create space for presence; AI-mediated triage can support prudent reasoning or impose opaque directives. DiRePal’s “architecture of prudence” translates Tronto’s insights into design principles for digital systems: contestability, reversibility, traceability, and temporal proportionality must be embedded into algorithmic workflows to ensure that technology remains subordinate to human dignity (Floridi 2013; Dignum 2019).

### Toward a relational–temporal ontology of dignity

Taken together, these philosophical traditions support a composite ontology of dignity grounded in 3 claims:

1. **Dignity is hermeneutic and diachronic** (Ricoeur): it requires kairotic time and interpretive space for re-narration, hosting fragmentary or symbolic expressions of selfhood.
2. **Dignity is ethical and asymmetrical** (Levinas): it arises from responsibility toward the vulnerable Other, not merely from autonomy or narrative capacity.
3. **Dignity is capability-dependent and structurally mediated** (Nussbaum; Tronto): it is sustained by enabling conditions – organizational, technological, relational – that support agency, affiliation, and recognition.

From these claims follow 2 operational corollaries central to DiRePal. The **temporal dignity indicator** reframes time allocation as an ethical metric – the ratio of dialogical presence to screen-facing tasks. The **architecture of prudence** reframes digitalization as a site of moral design, requiring algorithms to remain contestable, reversible, and temporally proportionate (Floridi 2013; Dignum 2019; Topol 2019).

These foundations also clarify the inclusion of a **post-biographical horizon of dignity**. When speech ceases or life ends, dignity persists as a claim of memory, recognition and ethical stewardship – how bodies are honored, names preserved, and digital traces governed. A relational–temporal account, therefore, extends care beyond biological life into the communal work of remembrance, resisting anonymity and affirming worth (Butler 2004; Guite-Verret et al. 2023).

Taken together, these philosophical traditions converge into a coherent relational–temporal ontology of dignity. Ricoeur provides the narrative and temporal structure through which identity is continuously reinterpreted; Levinas grounds dignity in ethical responsibility toward the vulnerable Other; Nussbaum situates dignity within conditions that enable or constrain human flourishing; and Tronto exposes the structural and institutional dimensions that sustain or undermine care. The DiRePal model emerges from this convergence, integrating these dimensions into a unified ethical grammar capable of addressing the complexities of contemporary, technologically mediated end-of-life care.

## The DiRePal model: Definition and structure

The DiRePal model brings together the philosophical foundations outlined above into a conceptual framework for understanding dignity in contemporary end-of-life care. Rather than offering a prescriptive protocol, DiRePal provides an ethical grammar – a way of perceiving, interpreting, and sustaining dignity as a relational and temporally dynamic phenomenon shaped by structure, context, and technological mediation. It positions dignity not as a static personal attribute but as something co-created through recognition, lived time, and shared narrative. Within this framework, dignity is not treated as a fixed property but as a relational achievement emerging through interaction, temporality, and structural conditions of care.

### Core axes of DiRePal

DiRePal is articulated around 3 interdependent axes that translate its philosophical foundations into clinical and institutional sensibilities: **Relational Recognition, Temporal Sensitivity**, and **Situated Co-Narrative**.

1. **Relational Recognition**

Dignity emerges in the encounter through attentiveness, presence, and the recognition of the other as irreducible. Such recognition is expressed not only in speech but also in silence, gesture, and naming. It encompasses patients, families, and clinicians, affirming the dignity of all participants in the caring relationship (Lévinas 1972; Rushton 2018).

1. **Temporal Sensitivity**

Across the illness trajectory, dignity fluctuates and requires openness to *kairotic* time – the singular, opportune moment of listening or presence. Temporal sensitivity resists the colonization of clinical encounters by chronometric metrics or algorithmic logic and requires institutional arrangements that protect time for narrative, interpretation, and human meaning (Ricoeur 1992; Tronto 1993).

1. **Situated Co-Narrative**

Dignity is sustained and reshaped through stories – linear or fragmented – co-authored between patients, families, and professionals. Co-narrative recognizes that meaning-making often occurs through shared testimony, gestures, rituals, or digital traces. Unlike the scripted structure of DT or the purely existential depth of presence, co-narrative offers a flexible, relational space where identity can be renegotiated in the face of illness (Butler 2004, 2005; Guite-Verret et al. 2023).

Together, these axes establish DiRePal as a grammar of dignity understood as event rather than possession, and as relational becoming rather than static essence.

DiRePal invites medicine to remember that, even in an algorithmic age, dignity is not measured but received – through presence, time, and shared recognition.

### Differences with prevailing models

DiRePal advances beyond existing models by addressing conceptual gaps left by both DT and the ethics of presence.

DT depends on structured interviews and coherent autobiographical recall; DiRePal instead welcomes fragmented, nonlinear, or collective storytelling, emphasizing interpretive re-narration rather than textual legacy (Ricoeur 1992).

The ethics of presence rightly centers attentiveness and listening but depends heavily on individual virtue and remains vulnerable to structural erosion. DiRePal embeds presence in institutional arrangements – supported by the temporal dignity indicator – ensuring that time-for-presence is protected rather than assumed (Brown-Johnson et al. 2019).

In doing so, DiRePal offers a broader ethical grammar that responds both to the reductionism of scripted interventions and to the idealization of purely interpersonal models. It brings relational, temporal, and structural dimensions together in a way that is responsive to contemporary clinical complexity.

### Operational concepts

Two operational concepts translate the DiRePal model into institutional practice and offer concrete handles for ethical implementation in clinical environments increasingly shaped by digital tools.

#### Temporal dignity indicator

This indicator measures the proportion of clinical time devoted to dialogical presence – listening, silence, shared attention – relative to time consumed by screens, alerts, and algorithmic tasks. It reframes time not as a logistical constraint but as an ethical resource and provides a tangible mechanism for institutional accountability (Topol 2019; Andersson et al. 2022). Importantly, this indicator does not imply that only direct patient-facing time contributes to dignity; rather, it recognizes that indirect clinical activities – such as clinical reasoning, symptom control, prescribing, and coordination of care – are also integral expressions of commitment to the patient’s well-being and dignity. Rather, the temporal dignity indicator is proposed as a heuristic and organizational tool aimed at preventing the structural marginalization of relational and dialogical presence within increasingly digitalized clinical environments.

### Architecture of prudence

Drawing on Aristotelian *phronesis* adapted to digital systems, the architecture of prudence embeds contestability, reversibility, traceability, and temporal proportionality into AI-mediated care. Algorithms must remain auditable and open to human revision; patients must retain access to explanation and second opinion; and technological mediation must remain proportionate to relational and ethical needs (Floridi 2013; Dignum 2019). These 2 tools express DiRePal’s commitment to ensuring that digital systems serve dignity rather than displace it.

No existing dignity framework offers operational instruments for protecting dignity through temporal allocation or structural governance. DiRePal is, therefore, the first model to articulate dignity not only philosophically but also through concrete tools that safeguard relational presence and prudential oversight in digitalized care.

### Clinical illustration

Consider a patient with advanced lung cancer, fluctuating cognition, and limited capacity to generate a coherent narrative. Within DT, such a patient may be excluded due to the demands of structured storytelling. In DiRePal, however, dignity can be sustained through brief narrative fragments captured through ePROMs, complemented by family testimony and gestures of recognition by clinicians. The temporal dignity indicator ensures that time-for-presence is not overshadowed by digital workflow, and the architecture of prudence guarantees that AI-derived prognostic outputs remain contestable and secondary to clinical judgment.

This illustration shows how DiRePal reframes dignity as an evolving, co-constructed, and institutionally supported practice rather than the by-product of narrative coherence or singular presence. It foregrounds relational and temporal processes while accommodating technological mediation within an ethical horizon.

A second illustration concerns a non-verbal patient with advanced neurodegenerative disease whose family members disagree about escalating technological interventions. One group request aggressive monitoring based on algorithmic predictions of potential deterioration, while another prioritizes comfort, presence, and minimizing digital mediation. Within DiRePal, dignity is not determined by decisional capacity but by relational and temporal recognition. Care teams would, therefore, privilege kairotic time – attentive presence at the bedside – and use digital projections only as dialogical prompts rather than directives. The temporal dignity indicator would ensure that clinician time is anchored in human encounter rather than in digital surveillance, while the architecture of prudence would require that algorithmic alerts remain contestable, reversible, and proportionate to the patient’s relational and embodied context. This scenario illustrates how DiRePal orients difficult decisions not toward technological escalation or withdrawal but toward the preservation of dignity as a co-created and temporally unfolding practice.

## Post-biographical dignity and the ethics of memory

Most dignity frameworks focus primarily on the patient’s lived, biographical existence. The DiRePal model extends this horizon by introducing the notion of *post-biographical dignity*, recognizing that dignity does not end with the cessation of consciousness or biological life. Rather, it persists as a claim of recognition expressed through memory, mourning, and digital legacy. This extension is especially relevant in the algorithmic age, where the self continues to exist through data traces, archives, and digital representations long after death (Buitelaar 2017; Öhman et al. 2017).

### Dignity beyond the biographical self

Ricoeur emphasizes that identity is always mediated by narrative and shaped by the interpretations of others. When self-narration becomes impossible in dying, dignity becomes radically dependent on external narrators – families, clinicians, and communities – who preserve, interpret, and honor the patient’s story (Ricoeur 1992). J. Butler adds that *grievability*, the extent to which a life is considered worthy of mourning, is a central ethical measure of dignity. To be forgotten, anonymized, or reduced to a datapoint is to be denied dignity in the post-biographical realm (Butler 2004).

This perspective highlights the moral importance of remembrance rituals, naming practices, and symbolic gestures of continuity. It also exposes structural injustices: some lives remain less “grievable” due to systemic inequities affecting migrants, socioeconomically marginalized groups, or those without access to well-resourced healthcare (Walter 1996). Dignity-preserving care, therefore, cannot end at death. It requires an aftercare of memory that prevents persons from being consigned to anonymity once biological life ceases.

### Digital legacy and the algorithmic afterlife

In contemporary practice, post-biographical dignity is increasingly mediated by digital environments. Patients leave behind not only embodied memories but also extensive data traces – electronic health records, ePROM responses, social media archives, audio recordings, photographs, and digital-legacy platforms (Brubaker et al. 2013; Savin-Baden et al. 2020). These traces can provide comfort and continuity for families, but they also raise urgent ethical questions concerning consent, control, representation, and potential misuse.

Digital ethics scholars warn against the commodification of the dead and the exploitation of personal data for purposes misaligned with the person’s values (Öhman et al. 2018). The emerging “algorithmic afterlife” can become a site of injustice: identities reconstructed without consent, digital remains harvested for commercial gain, or individuals effectively excluded from digital continuity due to inequities in access.

For these reasons, DiRePal calls for a prudential governance of digital legacy. Ethical care requires that digital remains be handled with consent, respect, and alignment with the patient’s values. Just as corporeal remains are treated with symbolic care, so too must digital traces be stewarded responsibly. Extending dignity beyond biological life thus becomes a matter of relational and temporal responsibility for the traces a person leaves behind.

### Memory as relational continuity

Tony Walter’s work shows that modern death is mediated through communal practices of remembrance. Families, clinicians, and institutions co-author the continuing bonds that endure beyond the final breath (Walter 1996). In this light, post-biographical dignity becomes a relational practice enacted through how we recall, narrate, honor, and symbolically sustain the memory of those who have died.

This has direct implications for clinical practice and education. Professionals must be prepared not only to accompany the dying but also to support the bereaved, recognizing that dignity extends into the domain of memory. Institutional rituals – memorial services, remembrance spaces, anniversary acknowledgements, or digital-legacy practices – embed this continuity into the fabric of care.

For DiRePal, relationality is not interrupted by death. Care continues as an ethical obligation of memory and presence, affirming that dignity transcends the boundaries of biography. In this perspective, palliative care is not merely the management of dying but the cultivation of a narrative community that safeguards dignity even when voices fall silent.

## Clinical practice in the algorithmic age: AI, ePROMs, and relational compression

Contemporary clinical practice increasingly unfolds within a digital environment shaped by AI, predictive analytics, and ePROMs. While these tools promise efficiency, anticipatory care, and greater personalization, they also risk creating a form of relational compression – transforming the clinical encounter into a site of data extraction and algorithmic interpretation. For the DiRePal model, this is not a peripheral concern but a central ethical challenge: if dignity is relational and temporal, technologies that reshape time, attention, and narrative space become ethically decisive.

### Potentials and risks of AI in end-of-life care

AI systems are increasingly used for prognostication, triage, and referral within palliative pathways (Heinzen et al. 2023; Wilson et al. 2023). Their statistical logic, however, is oriented toward population-level regularities rather than singular narratives. Even when technically robust, such systems can displace *kairotic* clinical time with chronometric predictions (Floridi 2013; Topol 2019). Two major risks follow from a DiRePal perspective. First, patients may be re-identified primarily as algorithmic risk profiles, narrowing the perception of their lived experience. Second, clinicians may become supervisors of machine recommendations rather than interpreters of human stories, eroding the prudential reasoning (*phronesis*) essential in end-of-life care.

Justice concerns compound these issues. Algorithmic systems can reproduce or amplify inequities encoded in their training data, influencing access to palliative services – an effect already documented in health-system algorithms outside palliative care (Obermeyer et al. 2019). These risks demand explicit governance. For DiRePal, the ethical risk is not only technical but phenomenological: suffering must remain grounded in the patient’s embodied, relational experience.

When AI reframes distress as a risk category, the model reorients interpretation back to the lived reality of the dying person, resisting the displacement of suffering by data-driven abstraction.

DiRePal’s architecture of prudence calls for institutional safeguards such as (i) guaranteed human review and second-opinion pathways; (ii) auditable model lineage and transparent updates; (iii) “pause” or opt-out functions when algorithmic outputs conflict with relational or temporal priorities; and (iv) clear role boundaries ensuring that AI remains advisory rather than directive in intimate end-of-life decisions. Far from rejecting technology, this stance subordinates it to an ethic of presence, recognition, and prudent timing.

### ePROMs: Amplifying or reducing ethical listening

When used well, ePROMs can improve symptom detection, quality of life, and timely intervention (Howell et al. 2015; Greenhalgh et al. 2018). Randomized and pragmatic studies show that structured symptom self-reporting may even improve survival in oncology (Denis et al. 2019), while trials of PRO-CTCAE confirm the feasibility of patient reporting in complex clinical contexts (Basch et al. 2017). Yet their ethical role depends on how they are integrated into clinical practice.

If treated as checklists or completed by clinicians instead of patients, ePROMs risk flattening existential concerns into binary responses and eroding the patient’s narrative voice (Snyder et al. 2009). DiRePal interprets ePROMs as potential dignity triggers: prompts that reveal hidden suffering, invite elaboration, and legitimize expression. This requires relational time, training, and narrative sensitivity – all supported by the temporal dignity indicator, which reframes clinical time as an ethical resource.

Practically, this means that ePROM collection should not consume more time in screen interaction than it returns in listening; results should be discussed with patients and families rather than silently archived; and alert thresholds should trigger relational responses – conversation, shared meaning-making – rather than purely transactional escalation (Al Sayah et al. 2021; Giesinger et al. 2021; Johnston et al. 2023).

### Integrating technologies within DiRePal

For DiRePal, the question is never simply whether to use AI or ePROMs, but how to integrate them ethically within a relational–temporal framework. Three principles follow:

1. **Dialogical mediation**

ePROMs must function as openings for narrative, not replacements for it; AI outputs must be explained in accessible language that invites participation, preserves the possibility of dissent, and recognizes silence as meaningful.

1. **Institutional embedding**

Safeguards such as human-in-the-loop review, audit trails, proportionate alert thresholds, and ethics oversight must be built into digital infrastructures (Dignum 2019; Topol 2019). These are ethical necessities, not technical luxuries.

1. **Prudential proportionality**

The degree of reliance on digital tools should vary with context. In large-scale triage, algorithms may support distributive justice; in intimate end-of-life conversations, human presence must predominate (Topol 2019).

Operationally, this means redesigning workflows around dignity: scheduling ePROM review within consultations; presenting dashboards that foreground narrative cues rather than only numerical scores; and configuring AI tools to default to explainable outputs and relational decelerators (e.g., “discuss before order”). Technologies that displace relational time compromise dignity; technologies that create time for presence become allies of DiRePal.

## Practical, educational, and institutional implications

The DiRePal framework is not merely a theoretical account but an ethical grammar capable of guiding clinical practice, professional training, and institutional design. By reframing dignity as relational, temporal, and structurally mediated, it generates practical implications across 3 interconnected domains: clinical care, education, and organizational policy.

### Clinical practice

For clinicians, DiRePal reframes dignity as a task grounded in presence and narrative hospitality. This involves 3 essential commitments.

First, **prioritizing time-for-presence**: relational listening must not be overshadowed by digital interfaces or throughput pressures. The temporal dignity indicator supports clinicians in protecting *kairotic* time, reframing clinical time from a neutral commodity into an ethical resource (Topol 2019).

Second, **co-narrative care**: interventions should include the voices of families and caregivers, moving beyond strictly dyadic models. Structured moments for shared storytelling – oral, written, or digital – affirm dignity as intersubjective and co-authored (Guite-Verret et al. 2023).

Third, **dialogical integration of digital tools**: ePROMs and AI systems must serve as prompts for deeper narrative exploration rather than mechanistic endpoints. When handled dialogically, digital outputs can enrich, rather than erode, ethical listening (Greenhalgh et al. 2018).

### Professional education

Palliative care education has traditionally emphasized technical competence and symptom management. DiRePal argues for a complementary emphasis on relational and ethical competencies.

Curricula should include narrative ethics, relational communication, moral resilience, and literacy in digital dignity. Clinicians must be prepared not only to interpret symptoms but also stories, silences, and digital traces as elements of dignity-preserving care (Rushton 2018; Andersson et al. 2022).

Pedagogically, reflective writing, narrative supervision, simulations of relational scenarios, and interdisciplinary ethics rounds help cultivate attentiveness to relational–temporal dynamics (Brown-Johnson et al. 2019).

Finally, **moral resilience** is essential: professionals need tools to protect their own dignity, recognizing that patient dignity is inseparable from the moral well-being of those who care for them (Rushton 2018).

### Institutional and policy implications

Institutions are not neutral backdrops but active mediators of dignity. The DiRePal framework underscores the need for organizational structures that protect relational and temporal recognition.

The **architecture of prudence** calls for embedding contestability, reversibility, traceability, and proportionality into digital infrastructures, ensuring technology enhances rather than colonizes care (Tronto 1993; Dignum 2019).

**Quality standards** should incorporate indicators of relational and temporal care – time spent in presence, patient and family feedback, and measures of staff moral well-being – within accreditation and quality improvement programs (Johnston et al. 2023).

**Ethical leadership** is also essential: leaders must cultivate cultures that value presence and narrative work, incorporating relational competence into recruitment, evaluation, and reward structures (Guite-Verret et al. 2023).

Institutions should also develop **rituals of remembrance** – memorial services, farewell rituals, curated digital legacies – to honor post-biographical dignity and ensure that patients’ lives remain recognized beyond biological existence (Walter 1996; Brubaker et al. 2013).

### Toward systemic embedding

In sum, DiRePal reframes dignity not as a fragile individual attribute but as a systemic ethical mandate. By recalibrating clinical practice, educational priorities, and institutional policies, it offers a framework capable of resisting the reduction of care to efficiency metrics and re-centering healthcare on relational and temporal recognition. Dignity, in this perspective, is not preserved solely in singular encounters but must be supported by the structures in which care unfolds. This systemic embedding ensures that the ethical grammar of DiRePal informs not only bedside decisions but also the cultural ethos and institutional architectures that shape contemporary palliative care.

## Objections and responses

No ethical framework is immune to critique. Anticipating objections strengthens the internal coherence of DiRePal and clarifies its distinctiveness within end-of-life ethics. Three recurrent concerns deserve attention: perceived philosophical abstraction, questions of empirical grounding, and potential overlap with existing dignity frameworks.

### “The model is too philosophical and impractical”

**Objection.** Some may argue that DiRePal relies heavily on complex philosophical sources and does not offer sufficiently concrete guidance for clinicians working under time pressure. In environments where clarity and immediacy are essential, philosophical nuance may appear distant from bedside practice.

**Response.** DiRePal is not presented as a metaphysical doctrine but as a pragmatic ethical grammar. Its conceptual foundations translate directly into operational tools such as the temporal dignity indicator and the architecture of prudence. These tools provide measurable, actionable metrics: the former reframes relational time allocation as an ethical priority, while the latter embeds contestability and reversibility into digital infrastructures. Rather than adding burden, DiRePal equips clinicians and organizations with ethical instruments for navigating technologically mediated care (Dignum 2019; Topol 2019). Its purpose is precisely to make philosophical insight clinically usable.

### “The framework lacks empirical validation”

**Objection.** A further concern is that, without large-scale empirical testing, DiRePal may appear speculative when compared with established interventions such as DT (Chochinov et al. 2011).

**Response.** DiRePal is explicitly offered as a philosophical–ethical framework, not as an intervention protocol. Its role is to provide the conceptual scaffolding within which empirical research can unfold. Indeed, the model is currently being operationalized through focus groups with patients, caregivers, and clinicians to examine relational and temporal dimensions of dignity in real-world settings (Johnston et al. 2023). Moreover, DiRePal integrates empirical insights already demonstrated in related fields – for example, the impact of ePROMs on quality of life and communication (Greenhalgh et al. 2018; Denis et al. 2019). Instead of competing with empirical inquiry, DiRePal offers a reflective horizon that enables such findings to be interpreted ethically and coherently.

### “It overlaps with dignity therapy or the ethics of presence”

**Objection.** Given its focus on dignity, DiRePal could be seen as a reformulation of existing models, particularly DT or Julião’s ethics of presence.

**Response.** DiRePal acknowledges its debt to these traditions but intentionally advances beyond them. Its contribution is 3-fold.

First, **relational breadth**: whereas DT foregrounds the patient’s voice, DiRePal includes family and professional dignity as integral elements of the ethical ecology of care (Rushton 2018).

Second, **temporal depth**: DiRePal situates dignity within temporal fluctuation, requiring sustained attentiveness across illness trajectories rather than relying on scripted interventions or isolated existential moments (Ricoeur 1992).

Third, **structural integration**: unlike DT or presence-based models, DiRePal directly addresses systemic and technological mediations, offering institutional principles – contestability, reversibility, proportionality – for the algorithmic age (Brubaker et al. 2013; Floridi 2013; Öhman et al. 2018).

In this sense, DiRePal is not a competitor but a philosophical evolution. It deepens the theoretical grounding of dignity, broadens its relational scope, and aligns it with the ethical challenges of contemporary digital medicine.

## Conclusion

Dignity at the end of life has long been recognized as an indispensable yet fragile value. Existing frameworks – from Chochinov’s DT to Julião’s ethics of presence – have enriched clinical and ethical discourse, but their limitations become more apparent in contemporary practice: the former through its reliance on coherent autobiography and individual narrative continuity, the latter through its idealization of presence and lack of systemic integration. In an era where clinical encounters are increasingly mediated by AI, ePROMs, and digital infrastructures, these limitations are sharpened. Without renewed philosophical grounding, dignity risks erosion through metrics, temporal compression, and the algorithmic handling of memory and identity.

The DiRePal model responds to these challenges by reconfiguring dignity as relational, temporal, and structurally mediated. Drawing on Ricoeur’s hermeneutics of narrative identity, Levinas’s ethics of alterity, Nussbaum’s capabilities approach, and Tronto’s care ethics, DiRePal understands dignity not as a static possession but as a dynamic event. It emerges through relationships, fluctuates across the illness trajectory, and is sustained – or undermined – by systemic and technological practices. Its operational innovations – the temporal dignity indicator and the architecture of prudence – translate philosophical insight into concrete institutional principles, enabling the protection of relational presence and the embedding of contestability, reversibility, and proportionality within digital systems.

DiRePal also extends the moral horizon of dignity beyond the biographical self. By incorporating the notion of post-biographical dignity, it highlights memory, grievability, and digital legacy as ethical dimensions of care. This extension resists the anonymity that can follow death in technologically mediated societies, ensuring that dignity continues through remembrance and narrative continuity, not only through biological life. By clarifying dignity as a relational, temporal, and structurally mediated phenomenon, DiRePal also addresses longstanding ambiguities in its conceptualization, offering a more precise and clinically meaningful framework for ethical reflection and practice.

Rather than competing with existing dignity frameworks, DiRePal offers a philosophical evolution. It provides a comprehensive ethical grammar that integrates relational, temporal, and post-biographical dimensions while addressing the ethical tensions introduced by digitalization. Its contribution lies in bridging philosophy and practice, offering clinicians, educators, and institutions a conceptual compass for resisting relational compression and cultivating care that is attentive, prudent, and just.

At stake is not only the dignity of dying patients but also that of caregivers, families and communities. By reframing dignity in relational–temporal terms, DiRePal offers a horizon of ethical resilience for 21st-century medicine – one that makes time, protects memory, and ensures that even in the algorithmic age, care remains a profoundly human act.

## References

[ref1] Al Sayah F, Lahtinen M, Bonsel GJ, et al. (2021) A multi-level approach for the use of routinely collected patient-reported outcome measures (PROMs) data in healthcare systems. *Journal of Patient-Reported Outcomes* 5(Suppl 2), 98. 34637031 10.1186/s41687-021-00375-1 PMC8511251

[ref2] Andersson H, Svensson A, Frank C, et al. (2022) Ethics education to support ethical competence learning in healthcare: An integrative systematic review. *BMC Medical Ethics* 23(1), 29. 35305627 10.1186/s12910-022-00766-z PMC8933936

[ref3] Aristotle (2009) *The Nicomachean Ethics*. Oxford: Oxford University Press.

[ref4] Basch E, Deal AM, Dueck AC, et al. (2017) Overall survival results of a trial assessing patient-reported outcomes for symptom monitoring during routine cancer treatment. *Jama* 318(2), 197–198. 28586821 10.1001/jama.2017.7156 PMC5817466

[ref5] Brown-Johnson C, Schwartz R, Maitra A, et al. (2019) What is clinician presence? A qualitative interview study comparing physician and non-physician insights about practices of human connection. *BMJ Open* 9(11), e030831. 10.1136/bmjopen-2019-030831 PMC6858153 31685506

[ref6] Brubaker JR, Hayes GR and Dourish P (2013) Beyond the grave: Facebook as a site for the expansion of death and mourning. *The Information Society* 29(3), 152–163.

[ref7] Buitelaar JC (2017) Post-mortem privacy and informational self-determination. *Ethics and Information Technology* 19(2), 129–142.

[ref8] Butler J (2004) Precarious Life: The Powers of Mourning and Violence.

[ref9] Butler J (2005) *Giving an Account of Oneself*. New York: Fordham University Press.

[ref10] Canguilhem G (2022) The normal and the pathological. In Canguilhem G (ed), *Knowledge of Life*. New York: Fordham University Press, 121–133.

[ref11] Chochinov HM, Cann B, Cullihall K, et al. (2012) Dignity therapy: A feasibility study of elders in long-term care. *Palliative and Supportive Care* 10(1), 3–15. 22329932 10.1017/S1478951511000538

[ref12] Chochinov HM, Hack T, Hassard T, et al. (2005) Dignity therapy: A novel psychotherapeutic intervention for patients near the end of life. *Journal of Clinical Oncology* 23(24), 5520–5525. 16110012 10.1200/JCO.2005.08.391

[ref13] Chochinov HM, Kristjanson LJ, Breitbart W, et al. (2011) Effect of dignity therapy on distress and end-of-life experience in terminally ill patients: A randomised controlled trial. *The Lancet Oncology* 12(8), 753–762. 21741309 10.1016/S1470-2045(11)70153-X PMC3185066

[ref14] Denis F, Basch E, Septans AL, et al. (2019) Two-year survival comparing web-based symptom monitoring vs routine surveillance following treatment for lung cancer. *Jama* 321(3), 306–307. 30667494 10.1001/jama.2018.18085 PMC6439676

[ref15] Dignum V (2019) *Responsible Artificial Intelligence: How to Develop and Use AI in a Responsible Way*. Cham: Springer.

[ref16] Floridi L (2013) *The Ethics of Information*. UK: Oxford University Press.

[ref17] Foucault M (1980) *The History of Sexuality. Volume One: An Introduction*. New York: Vintage Books.

[ref18] García Abejas A, Santos DG, Mota-Filipe H, et al. (2025) Post-biographical dignity in the age of artificial intelligence: Narrative, ePROMs and ethical challenges in end-of-life care. *Palliative and Supportive Care* 23, e192. 41143393 10.1017/S1478951525100990 PMC13166595

[ref19] Giesinger JM, Efficace F, Aaronson N, et al. (2021) Past and current practice of patient-reported outcome measurement in randomized cancer clinical trials: A systematic review. *Value in Health* 24(4), 585–591. 33840437 10.1016/j.jval.2020.11.004

[ref20] Greenhalgh J, Gooding K, Gibbons E, et al. (2018) How do patient reported outcome measures (PROMs) support clinician-patient communication and patient care? A realist synthesis. *Journal of Patient-Reported Outcomes* 2(1), 42. 30294712 10.1186/s41687-018-0061-6 PMC6153194

[ref21] Guite-Verret A, Vachon M and Girard D (2023) Intentional presence and the accompaniment of dying patients. *Medicine, Health Care and Philosophy* 26(3), 477–486. 37338776 10.1007/s11019-023-10161-z PMC10425290

[ref22] Heinzen EP, Wilson PM, Storlie CB, et al. (2023) Impact of a machine learning algorithm on time to palliative care in a primary care population: Protocol for a stepped-wedge pragmatic randomized trial. *BMC Palliative Care* 22(1), 9. 36737744 10.1186/s12904-022-01113-0 PMC9896817

[ref23] Howell D, Molloy S, Wilkinson K, et al. (2015) Patient-reported outcomes in routine cancer clinical practice: A scoping review of use, impact on health outcomes, and implementation factors. *Annals of Oncology* 26(9), 1846–1858. 25888610 10.1093/annonc/mdv181

[ref24] Johnston B, Donmez CF and Juliao M (2023) Effectiveness of dignity therapy in the context of culturally competent care in people with palliative care needs: A systematic review of systematic reviews. *Current Opinion in Supportive and Palliative Care* 17(3), 186–192. 37428208 10.1097/SPC.0000000000000664

[ref25] Juliao M, Johnston B and Antunes B (2022) Dignity Therapy - Past, present and future journey: Beyond end of life cancer care. Responding to Grassi et al. *Psychooncology* 31(8), 1431–1432. 35719045 10.1002/pon.5981

[ref26] Juliao M, Oliveira F, Nunes B, et al. (2017) Effect of dignity therapy on end-of-life psychological distress in terminally ill Portuguese patients: A randomized controlled trial. *Palliative and Supportive Care* 15(6), 628–637. 28166861 10.1017/S1478951516001140

[ref27] Kant I (2011) *Groundwork of the Metaphysics of Morals: A German-English Edition*. Cambridge: Cambridge University Press.

[ref28] Lévinas E (1972) *Humanisme de L’autre Homme*. Montpellier: Fata Morgana.

[ref29] Lévinas E and Nemo P (1985) *Ethics and Infinity*. Pittsburgh: Duquesne University Press.

[ref30] Nussbaum MC (2011) *Creating Capabilities: The Human Development Approach*. Cambridge, MA: Harvard University Press.

[ref31] Obermeyer Z, Powers B, Vogeli C, et al. (2019) Dissecting racial bias in an algorithm used to manage the health of populations. *Science* 366(6464), 447–453. 31649194 10.1126/science.aax2342

[ref32] Öhman C and Floridi L (2017) The Political Economy of Death in the Age of Information: A Critical Approach to the Digital Afterlife Industry. *Minds & Machines* 27(4), 639–662. doi:10.1007/s11023-017-9445-2

[ref33] Öhman C and Floridi L (2018) An ethical framework for the digital afterlife industry. *Nature Human Behaviour* 2(5), 318–320. doi:10.1038/s41562-018-0335-2 30962597

[ref34] Ricoeur P (1991) Narrative Identity. *Philosophy Today* 35(1), 73–81.

[ref35] Ricoeur P (1992) *Oneself as Another*. Chicago: University of Chicago Press.

[ref36] Rushton CH (2018) Moral resilience: Transforming moral suffering in health care. New York: Oxford University Press.

[ref37] Savin-Baden M. and Mason-Robbie V (2020) *Digital afterlife: Death matters in a digital age*. Boca Raton: CRC Press.

[ref38] Snyder CF and Aaronson NK (2009) Use of patient-reported outcomes in clinical practice. *The Lancet* 374(9687), 369–370. doi:10.1016/S0140-6736(09)61400-8 19647598

[ref39] Topol E (2019) *Deep Medicine: How Artificial Intelligence Can Make Healthcare Human Again*. New York: Basic Books.

[ref40] Tronto J (1993) *Moral Boundaries: A Political Argument for an Ethic of Care*. New York: Routledge.

[ref41] Tronto JC (2013) *Caring Democracy Markets, Equality, and Justice*. New York: New York University Press.

[ref42] Walter T (1996) A new model of grief: Bereavement and biography. *Mortality* 1(1), 7–25.

[ref43] Webster JD and Haight BK (2002) *Critical Advances in Reminiscence Work: From Theory to Application*. New York, NY, US: Springer Publishing Company.

[ref44] Wilson PM, Ramar P, Philpot LM, et al. (2023) Effect of an artificial intelligence decision support tool on palliative care referral in hospitalized patients: A randomized clinical trial. *Journal of Pain and Symptom Management* 66(1), 24–32. 36842541 10.1016/j.jpainsymman.2023.02.317

